# Uncovering the mechanisms of research capacity development in health and social care: a realist synthesis

**DOI:** 10.1186/s12961-018-0363-4

**Published:** 2018-09-21

**Authors:** Jo Cooke, Paolo Gardois, Andrew Booth

**Affiliations:** 1 NIHR CLAHRC Yorkshire & Humber, Research Capacity and Engagement Programme Management, 11 Broomfield Road, Sheffield, S10 2SE United Kingdom; 2 Department of Public Health and Pediatrics, University of Turin, Turin, Italy; 3 School of Health and Related Research, University of Sheffield, Regent Court, 30 Regent Street, Sheffield, S1 4DA United Kingdom

**Keywords:** Research capacity development, Realist synthesis, Evaluation, Leadership, Training

## Abstract

**Background:**

Research capacity development (RCD) is considered fundamental to closing the evidence–practice gap, thereby contributing to health, wealth and knowledge for practice. Numerous frameworks and models have been proposed for RCD, but there is little evidence of what works for whom and under what circumstances. There is a need to identify mechanisms by which candidate interventions or clusters of interventions might achieve RCD and contribute to societal impact, thereby proving meaningful to stakeholders.

**Methods:**

A realist synthesis was used to develop programme theories for RCD. Structured database searches were conducted across seven databases to identify papers examining RCD in a health or social care context (1998–2013). In addition, citation searches for 10 key articles (citation pearls) were conducted across Google Scholar and Web of Science. Of 214 included articles, 116 reported on specific interventions or initiatives or their evaluation. The remaining 98 articles were discussion papers or explicitly sought to make a theoretical contribution. A core set of 36 RCD theoretical and conceptual papers were selected and analysed to generate mechanisms that map across macro contexts (individual, team, organisational, network). Data were extracted by means of ‘If-Then’ statements into an Excel spreadsheet. Models and frameworks were deconstructed into their original elements.

**Results:**

Eight overarching programme theories were identified featuring mechanisms that were triggered across multiple contexts. Three of these fulfilled a symbolic role in signalling the importance of RCD (e.g. positive role models, signal importance, make a difference), whilst the remainder were more functional (e.g. liberate talents, release resource, exceed sum of parts, learning by doing and co-production of knowledge). Outcomes from one mechanism produced changes in context to stimulate mechanisms in other activities. The eight programme theories were validated with findings from 10 systematic reviews (2014–2017).

**Conclusions:**

This realist synthesis is the starting point for constructing an RCD framework shaped by these programme theories. Future work is required to further test and refine these findings against empirical data from intervention studies.

**Electronic supplementary material:**

The online version of this article (10.1186/s12961-018-0363-4) contains supplementary material, which is available to authorized users.

## Background

National policy and financial investment across the globe indicates overwhelming support for building research capacity in healthcare systems. Enhanced capacity is believed to promote problem solving [1], reduce the gap between evidence and practice [2, 3], and promote health gains [4]. It is considered a powerful and cost-effective way of advancing healthcare and development [5] and, if done well, can improve collaboration between high- and low-income countries [1] and address health inequalities [6]. United Kingdom policy highlights that the ‘best’ health research promotes the health and wealth of the nation [7].

Despite overwhelming support in the research literature and policy documents, research capacity development (RCD) is poorly defined, and conceptually elusive [8]. Undertaking a conceptual review of the literature, Condell and Begley define research capacity-building, a component of RCD, as “*a funded, dynamic intervention operationalised through a range of foci and levels to augment ability to carry out research or achieve objectives in the field of research over the long-term, with aspects of social change as an ultimate outcome*” [8]. This definition highlights that RCD is complex and operates at a number of structural levels, including individual, team, organisational and within networks [9], and includes a range of ‘interventions’ [10] or foci of activity. RCD activities are often conducted in parallel and can be interrelated. Research training, fellowships and mentorship schemes, for example, can be planned and evaluated separately, but in practice are often linked [11] and add value to each other. RCD ‘interventions’ may include processes such as priority-setting [12], but equally can incorporate structural changes in organisations, for example, developing an information technology infrastructure [13, 14]. Structural and process interventions can link to outcomes of their own but can also produce a fertile environment for other RCD initiatives. Collectively and separately, they achieve the common goal of stimulating ‘more research done well’.

The challenge of understanding RCD and how it operates is compounded by the fact that RCD is often seen as a means to an end [9] rather than an end in itself. From this perspective, means are the skills and structural enhancements that enable research to be conducted with the ultimate ‘end’ or goal of RCD in healthcare being to change practice and systems to improve health [13]. Setting the criteria for impact to this level makes it even more challenging to understand ‘what works’ in RCD. Although targeting improved health as the goal gives more value and ‘meaning’ to RCD activity, it necessitates long and convoluted causal chains, making it even more difficult to attribute impact. It also requires consideration of the nature and quality of how RCD interventions are performed, whether they are ‘meaningful’ and whether they can produce change in practice. Nuyens, for example, suggests that how priority-setting is undertaken influences how meaningful it is, and whether research is subsequently used in practice [15].

The literature on RCD reflects its elusive and chameleon-like quality and how difficult it is to measure and attribute impact [16]. Reports and case studies of RCD interventions in health do exist but evidence on effectiveness is inconclusive. Many evaluations fall short of being able to determine the impact on healthcare systems and thus demonstrate meaningful RCD. Such limitations may, in turn, reflect shortcomings in the theoretical underpinning of RCD interventions [16].

Further complexity is added when considering the relationship between RCD and knowledge mobilisation (KM). RCD focuses very much on empowering and enabling different levels of the health research system to conduct research. KM focuses further downstream – once the capacity is in place, to what extent can the research that has been generated change practice? Many health systems display an evolutionary or developmental aspect to these activities; in the United Kingdom, for example, antecedent investment in research and development support units has started to deliver the capacity to mobilise knowledge through networks such as the Collaborations for Leadership in Applied Health Research and Care (CLAHRCs). However, typically, RCD and KM co-exist and interact given that new requirements identified from KM will feed into subsequent requirements for specific reconfigurations of research capacity.

In summary, RCD can be categorised as a complex intervention, what Willis et al. describe as “*a set of purposefully coordinated components that target multiple levels and sectors of a system, that operate both independently and inter-dependently, and that interact in the contexts in which they are implemented*” [17]. Realist approaches offer an ideal methodology for understanding, evaluating and planning such interventions.

Numerous models suggest how RCD works [1, 9, 14, 18–21]. However, few models address underlying mechanisms for what works, and why, across a range of contexts, in achieving a meaningful impact on health services and systems. No attempt has been undertaken to link existing RCD models, nor to develop theories in a systematic way from them.

The objective of this realist synthesis is to address what are the mechanisms that support meaningful RCD that are triggered across diverse contexts, specifically at individual, team, organisation or network level, as described in conceptual and theoretical papers? We aim to isolate mechanisms that are activated across and within diverse contexts in order to develop programme theories that will identify and test causal chains in RCD programmes.

## Methods

This paper reports on a work package within a larger programme of work on reviewing the RCD literature in health and social care. The wider programme aims to analyse both the conceptual papers and intervention studies. This paper reports on the first stage of this research, namely a realist synthesis of theoretical and conceptual papers to develop programme theory at a macro level. A realist synthesis seeks to explain and unpack the mechanisms by which an intervention works (or fails to work); it seeks to advance a potential explanation, as opposed to a definitive judgement, about how interventions (in this case research capacity development activities) achieve their outcomes [22]. By mechanisms we mean the responses that the ‘active’ components within an intervention stimulate, either individually or collectively, within participants. We explore these mechanisms by first identifying an accessible explanation of how the intervention is understood to work, known as a ‘programme theory’, derived from the research literature, official documents or stakeholder explanations.

A realist approach recognises that the context within which an intervention is delivered is “*complex, multi-faceted and dynamic*” [23]. It challenges the assumption, implicit in conventional systematic review methodology, that the same intervention will work in the same way in different contexts. Realist logic seeks to articulate statements along the lines of ‘IF Context A includes… THEN Mechanisms X, Y, Z are activated LEADING TO Outcome O^1^’ – these statements are technically known as ‘CMO Configurations’ or ‘CMO chains’.

The work described in this study seeks to identify mechanisms that are activated across a variety of contexts operating at different levels (individual, team, organisational, network) within the health research system to achieve either desirable or unintended outcomes. Subsequent work will seek to map evidence from empirical studies to the theoretical framework. The realist synthesis method was supported by systematic mapping methodology. A core set of theoretical and conceptual papers on RCD was analysed in order to generate mechanisms and map these across macro contexts (individual, team, organisational, network) in order to develop programme theories.

### Initial scoping of the literature

In 2005, a scoping review, Re:Cap – Identifying the Evidence Base for Research Capacity Development in Health and Social Care [10], was commissioned by the National Coordinating Centre for Research Capacity Development, in partnership with the National Steering Committee of Research and Development Support Units, to “*identify, map, and describe the literature available to inform research capacity development (RCD) activities in health and social care, and to inform the work of RDSUs* [Research and Development Support Units]” (the then United Kingdom regional research and development support units) [10]. A scoping review is “*a preliminary assessment of the potential size and scope of the available research literature*” [24] and does not seek to conduct formal assessments of evidence quality. One component of the scoping review sought to identify existing RCD models, frameworks and theories. Four limitations were identified for this component of the wider scoping review, namely (1) models, frameworks and theories were derived from a heterogeneous range of sources and disciplines making comparison and specific application to health and social care problematic, especially as some models originated from outside the context of research capacity; (2) no recognised procedures existed for identifying models, frameworks and theories in a systematic way; (3) time constraints did not permit a formal attempt to examine an empirical base for each identified model, framework or theory; and (4) related to (3), there was limited opportunity to link the theories identified to eight RCD activities (Box 1) prioritised for the scoping review. Several years later, two authors of the original scoping review (JC & AB), working with a trained information specialist (PG), therefore sought to return to the topic area to consolidate opportunities identified from the quantity and characteristics of the literature in order to extend the conceptual thinking that underpins RCD.

A considerable body of literature describes models for RCD together with evaluations of specific individual RCD activities. However, none of the identified studies sought to explore beneath the level of actual interventions to examine the mechanisms by which interventions might achieve their intended effects. We hypothesised that some of these effects would be specific to the context of research capacity.

#### Search strategy

Structured database searches were conducted across seven databases (MEDLINE, EMBASE, ASSIA, CINAHL, ERIC, PsycInfo, and Web of Science) to identify papers that examined RCD in a health or social care context. Searches were conducted across the period 1998–2013. In addition, given the diversity of terminology, 10 key articles were selected from the original Re:CAP review [10] and designated as ‘citation pearls’. Citation searches were then conducted across Google Scholar and Web of Science for articles citing these conceptual works.

#### Inclusion/exclusion criteria

For inclusion in the initial review project a paper should:

1. Describe an RCD model/theory/framework OR
2. Evaluate a model/theory/framework cited from elsewhere OR
3. Report an evaluation of an intervention that was based on or cites model/theory/framework

AND

4. Be specific to a health or social care setting
5. Be published in English between 1999 and 2013

Note that subsequent phases of the project required validation and refreshment of the original dataset to extend coverage between 2014 and 2017. For details see below.

#### Keywords

The Research question was formulated according to the BeHeMoTH question structure [25]. This formulation is specifically designed to help to specify theory-related literature searches, as follows:

- **Be – Behaviour of Interest:** RCD (including eight specific interventions: Prioritisation; Mentoring for research; Research leadership; Research facilitation; Research skills training; Funding (including bursaries and fellowships); Networks and collaborations; Infrastructure).
- **H – Health Context:** Health and social care.
- **E – Exclusions:** Capacity development for other (non-research) purposes; Models of RCD not tried or proposed for a health and social care context.
- **MoTh – Models or Theories:** operationalised as a generic ‘model* or theor* or concept* or framework*’ strategy together with named models or theories if required. Illustrative keywords are given in Table 1.

**Table 1:** Summary of search strategies and search terms

Research		Capacity development		Health and social care		Models, etc.
Research	AND	‘capacity development’ OR ‘capacity building’ OR ‘capacity evaluation’ OR ‘community development’ OR ‘community building’ OR ‘building communities’	AND	Not specified on health/social care databases	AND	model* OR theor* OR concept* OR framework*
		prioritis* OR prioritiz* OR mentor* OR leader* OR facilitat* OR training OR OR funding OR bursaries OR fellowship* OR network* OR collaboration* OR infrastructure*		On non-health/social care databases: Health OR Nurs* OR Medical OR doctor OR paramedic* OR therapy OR therapist OR Physiotherap* OR ‘social work*’		
	NEAR/ SAME/ ADJ/ WITH^a^	‘capability’ OR ‘capacity’ OR ‘productivity’ OR ‘output’ OR ‘strategy’.				
Research capacity						
Researcher development						
Researcher career*						

^a^According to Database functionality

#### Key citation pearls

A list of 10 previous models identified for the Re:CAP project [1, 6, 9, 14, 18–21, 26, 27] (designated as ‘citation pearls’) was searched in 2014 using Google Scholar, Web of Science and Scopus. Supplementary search approaches proved of particular importance given the significant variation in terminology and the update role of the review. In addition, reference lists of included articles were examined for additional references not retrieved by the database keywords search. An update procedure was conducted, specifically for systematic reviews, in December 2017 (see the section Validation and refreshment of programme theories below).

#### Date and language restrictions

Papers were published between 2000 and 2015. Only English language papers were considered given the intended target audience for the review findings.

#### Quality assessment

No accepted instruments have been developed to assess the theoretical sufficiency of conceptual papers. Our overall goal was interpretative (configurative), not aggregative [28], so we did not exclude any studies based on study quality alone. However, we did examine included papers in relation to their perceived proximity to the United Kingdom context for which we were producing the review [29].

#### Data extraction

Data were extracted on author, year, country, context and, where appropriate, RCD activity and study type. For this work package, articles with RCD theories, models and frameworks were used to extract causative relations of components within them in the form of ‘If-Then’ statements. The level at which the activity was described as taking place was also extracted (individual, team, organisation and network level). These data were extracted into Excel sheets.

## Data synthesis

### Accessing the programme theories

Careful reading and re-reading of identified conceptual papers was undertaken individually by two investigators (AB, JC). Particular attention was focused on identifying causal chains by which an RCD programme or specific intervention might achieve either proximal (e.g. knowledge or skill gains) or long-term gains (i.e. improvements in health, increases in wealth or the achievement of an evidence-formed organisation or society). Data were extracted as If-Then statements into an Excel spreadsheet [30].

In cases of uncertainty, the If-Then statements, and proposed causal links, were discussed within the team. Chains of If-Then statements were constructed to yield insights on overall generic RCD approaches. A further complexity related to the level at which particular activities might be targeted, for example, at an individual, team, organisation or network level. If-Then statements were grouped by level of targeted intervention, with mechanisms that occurred at more than one contextual level being identified. In line with realist approaches, the team reasoned that similarities in mechanism, as opposed to the actual activities themselves, would extend across all levels of operations (context), to enable programme theory building.

### Validation and refreshment of programme theories

An essential feature of the realist synthesis method is the process of validating the original programme theories against a further independent dataset. Citation searches were repeated for the 10 citation pearls in December 2017 and all reviews published between 2014 and 2017 meeting the original inclusion criteria and exhibiting a recognisable degree of systematicity were analysed for confirmatory and original programme theory.

## Results

### Literature base

**Fig. 1:**
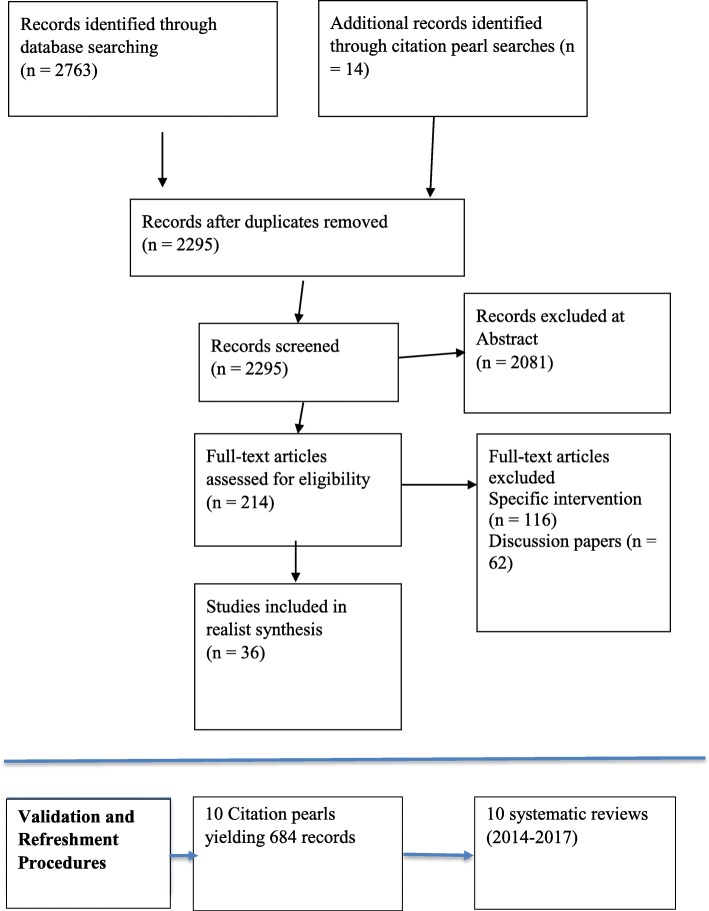
Flowchart for the systematic review following the PRISMA reporting methodology

The literature search was conducted across nine databases. Potentially relevant articles (*n* = 2763) were identified from searching electronic databases, and 14 additional potentially relevant articles were identified from follow-up of models and framework papers included in the earlier Re:CAP scoping review (the designated ‘citation pearls’). Of the potentially relevant articles, 2081 were excluded because their primary focus was not on RCD, leaving 682 potentially relevant papers from electronic databases; 468 articles were subsequently excluded at abstract stage, being either not relevant, only available in abstract form or written in a language other than English and 214 articles from electronic databases were retained, 116 of which reported on specific interventions or initiatives or their evaluation. A subset of 98 articles were discussion papers or explicitly sought to make a theoretical contribution. These 98 articles were read carefully by one of the review team (JC/AB) and, where the study was considered appropriate to the review question, selected for data extraction. A final list of 36 conceptual papers, reflecting consensus between the reviewers, were ultimately included. Figure 1 outlines the PRISMA diagram.

The 36 papers (Table 2) reflected international interest in RCD, with prominent players including the United Kingdom (*n* = 11), Australia (*n* = 6), the United States of America (*n* = 5), Canada [31] and Canada/United States combined [27] (North America). Five papers represented international perspectives, typically in the form of literature reviews. Low- and middle-income countries were represented by five collective papers and individual papers from Bangladesh [5], Liberia [20] and South Africa [21]. Primary care was the most prevalent field of research (*n* = 12), with nursing (*n* = 10), health and health services research (*n* = 9) also being well represented. Three papers examined allied health (*n* = 3) and the final paper looked at public health [21]. Collectively, the papers represented all the identified activities of RCD, with richer papers yielding programme theory relating to three or more activities (Table 2).

**Table 2:** The 36 studies with included research activities, aim of study and indicative If-Then statements

Author (Year) [Ref]	Context	Discipline	Included research activities	Aim of study	Indicative If-Thens
1. Albert & Mickan (2003) [4]	Australia	Primary Care	Training	To propose a paradigm shift in the content of capacity-building as a step towards closing the gaps between research, policy and practice	IF research ideas and implementation strategies are discussed and translated across several organisational contexts and cultures, THEN research influences practice
2. Breen et al. (2005) [21]^a^	South Africa	Public Health	Leadership, Networks, Resources, Training	To seek improved understanding of how RCB can be achieved and to propose a framework to improve understanding and delivery and to achieve better congruence between expectations and outcomes relating to RCB	IF investment is inadequate and incentives inappropriate, THEN organisations are unable to sustain RCD beyond the lifespan of a specific project
3. Coen et al. (2010) [31]	Canada	Health and Health Services Research	Infrastructure, Leadership, Networks	To explore potential for an expanded conceptualisation of research infrastructure, that specifies its largely assumed qualities whilst extending to articulate the interactive relationships among tangible and intangible systems and structures underlying centre functioning	IF organisational leaders develop an appropriate organisational research culture, THEN members collaborate on research IF researchers share a particular research identity, THEN researchers acquire a sense of belonging or a ‘ready-made affinity group’
4. Condell & Begley (2007) [8]	International	Nursing	Funding, Leadership, Training,	To conduct a concept analysis of capacity-building and its relationship to research	IF organisations engage in dynamic RCD activities, THEN organisations can achieve sustainability and ultimately effect social change
5. Conn et al. (2005) [49]	United States of America	Nursing	Funding, Prioritisation	To describe the success of one school of nursing in moving from having no NIH funding to being ranked in top 20 schools for NIH funding for consecutive years	IF staff in grant support services are not involved in graduate student education or research presentation materials, THEN this conveys a clear message about the importance of preparing competitive grant applications IF an organisation frequently communicates about grant activity, THEN the organisation cultivates an environment that is conducive to high research productivity
6. Cooke & Green (2000) [50]	International	Nursing Research	Prioritisation, Training	To identify factors that might affect the research capacity of departments of nursing in higher education, and to make recommendations to enable departments to develop their capacity to undertake research	IF nurse educators are encouraged to pursue further qualifications, particularly higher degrees, THEN teaching staff feel able to engage in research activity
7. Cooke et al. (2005) [9]^a^	United Kingdom	Primary Care	Networks, Training	To develop the debate around RCB by suggesting a framework for planning change and measuring progress, based on six principles of RCB	IF organisations support research ‘close to practice’, THEN stakeholders perceive that research is useful IF organisations develop linkages, partnerships and collaborations, THEN organisations can build up intellectual capital (knowledge) and social capital (relationships) IF organisations build up intellectual capital (knowledge) and social capital (relationships), THEN their ability to do research is enhanced IF research funders include continuity and sustainability in funding provision, THEN organisations can maintain and continue newly acquired skills and structures to undertake research
8. Cooke et al. (2015) [12]	United Kingdom	Health and Health Services Research	Funding, Leadership, Networks, Prioritisation, Training	To illustrate the use of collaborative priority-setting in a United Kingdom research collaboration (Collaboration and Leadership in Applied Health Research and Care – CLAHRC)	IF research networks identify ‘needs-led, meaningful’ research projects, THEN research is considered timely IF research networks harness flexible resources (people, funds, skills), THEN research can be responsive IF research leadership is responsive and transformative, THEN research can be co-produced
9. Del Mar & Askew (2004) [18]^a^	Australia	Primary Care	Funding, Networks, Training	To promote interventions for family/general practice RCB by describing successful international examples (e.g. diseases and illness research as well as process research); monitor output of research; increase number of research journals; encourage and enable research skills acquisition (including as part of professional training); strengthen academic base; and promote research networks and collaborations)	IF governments have family medicine research on their agendas (as shown by funding for RCB and for research activity itself), THEN governments send a clear message to clinical and academic communities that family medicine research is important and worthy of support
10. Edwards et al. (2009) [51]	LMICs	Nursing Research	Funding, Mentoring, Training	To identify long-standing barriers to nurses’ engagement in research and to discuss strategies to enable LMIC nurses to lead research of high relevance to local and international policy decisions affecting population health	IF researchers are given opportunities to work alongside senior researchers, both on-site and by distance, THEN they can discuss ways to balance research with teaching, clinical and administrative demands
11. Farmer & Weston (2002) [19]^a^	Australia	Primary Care	Funding, Mentoring, Networks	To propose a conceptual model to assist primary care RCB initiatives	IF research funders employ a whole system approach providing funding and resources at multiple levels, THEN practitioners can enter the system at an appropriate level, and then progress to a higher level of research capacity IF research funders accommodate diversity, THEN practitioners develop research interests in topics of ongoing personal interest IF research funders provide protected time for research, THEN individuals participate in research
12. Fenton et al. (2001, 2007) [52, 53]	United Kingdom	Primary Care	Networks	To reflect on the understanding of networks from organisational science and how this understanding can inform the development and evaluation of primary care research networks	IF researchers demonstrate socialisation, teamwork and openness, THEN researchers resist the tendency towards groupthink and open up opportunities for the exchange of ideas and knowledge
13. Fitzgerald et al. (2003) [54]	United States of America	Nursing	Mentoring, Networks	To discuss two paediatric critical care clinical nurse specialists’ participation in a collaborative research team led by university faculty	IF healthcare professionals are included as an integral part of the research team, THEN healthcare professionals receive mentoring in many aspects of the research process IF healthcare professionals are pulled away from their clinical unit to engage in research responsibilities, THEN other staff may resent the intrusion or see involvement in the project as frivolous when they are left with more work or without ready access to consultation
14. Gadsby (2011) [16]	LMICs	Health and Health Services Research	Funding, Networks, Training	To inform understanding of RCS, and how to consider the effectiveness of these initiatives by examining (1) understandings of and approaches to RCS, and (2) different ways in which RCS is monitored and evaluated	IF donors support individual capacity development at the expense of system capacity development, THEN individuals from LMICs leave for better jobs elsewhere
15. Golenko et al. (2012) [55]	Australia	Allied Health	Infrastructure, Leadership	To describe and analyse allied health senior manager perspectives of how organisational factors impact on RCB	IF staff are supported from a staff-time perspective to do research, THEN staff are motivated to participate in research IF staff receive recognition for research participation, THEN staff are motivated to participate in research
16. Green et al. (2007) [56]	United Kingdom	Nursing Research	Leadership, Networks, Training	To evaluate different approaches to RCD and to answer: ‘How do university departments develop the research capacity of their nursing/midwifery staff, what approaches do they use, and why are outcomes as they are?	IF researchers form alliances between novice and experienced researchers, THEN organisations achieve a balance between capacity development and leading-edge development
17. Jenerette et al. (2008) [6]^a^	United States of America	Nursing Research	Networks	To describe the models of research collaboration emerging from the Yale-Howard Partnership Center on Reducing Health Disparities by Self and Family Management	IF partners demonstrate effective communication and are sensitive to the history and unique characteristics of the partnering institution as well as its population, THEN investigators successfully complete projects on time and deliver subsequent presentations and publications
18. Johnson et al. (2005) [20]^a^	Liberia	Health and Health Services Research	Infrastructure, Training	To present an RCB model to strengthen HIV/AIDS service delivery system through a proposed Liberia–United States of America research partnership that focuses on establishing and strengthening HIV/AIDS service delivery system infrastructure and enhancing research and application skills of Liberian scientists and professionals	IF participant perceives the salience of the North–South partnership, THEN the participant is ready to participate in research IF participants are ready to participate in research, THEN their organisation sustains its research activities
19. Jones et al. (2003) [26]^a^	Australia	Primary Care	Training	To determine general practitioners’ research training needs and the barriers to involvement in research	IF GPs perceive that they do not possess the necessary research skills, THEN they are reluctant to engage in research
20. Lansang & Dennis (2004) [1]^a^	LMICs	Health and Health Services Research	Funding, Infrastructure, Mentoring, Networks, Training	To review the broad approaches taken to RCB and the likelihood that these efforts will prove sustainable	IF research funders promote ‘learning by doing’ approaches, such as developmental or seed grants, hands-on training in ongoing research programmes or mentorship programmes, THEN practitioners are encouraged to participate in research IF developing countries develop partnerships and networks with developed countries or other developing countries, THEN their collective outputs are greater than the sum of their isolated efforts
21. Levine et al. (2013) [13]	United States of America	Health and Health Services Research	Funding, Infrastructure, Mentoring, Networks, Training	To study two RCB programmes with similar goals and to expand upon the knowledge base of strategies and approaches to RCD and thus provide a better understanding of contextual factors that may influence the efficacy of RCD strategies	IF organisations develop good external and internal health services research partners, THEN they can build research capacity IF organisations are able to build on or leverage larger organisational changes, THEN they can achieve successful RCB
22. Macfarlane et al. (2005) [14]^a^	United Kingdom	Primary Care	Funding, Infrastructure, Leadership, Networks, Training	To identify key structural, developmental and environmental characteristics associated with successful and sustained involvement in research, and to inform national strategy for RCB in primary care	IF organisations produce a mission statement that acknowledges the value of research, THEN GPs develop a research practice
23. Mahmood et al. (2011) [5]	Bangladesh	Health and Health Services Research	Funding, Prioritisation	To identify problems faced by a health research institute in Bangladesh, describe two strategies developed to address these problems, and identify the results after 3 years of implementation	IF organisations develop a monitoring and evaluation framework, THEN donors do not exert an influence over organisational research priorities
24. Nchinda (2002) [57]	LMICs	Health and Health Services Research	Training	To describe some experiences in RCS over the last few decades and to propose, from these, mechanisms for sustainable RCB	IF returning researchers learn new skills and techniques when training overseas, THEN these researchers require access to appropriate equipment and resources when returning to their own institutions
25. North American Primary Care Research Group (2002) [27]^a^	North America	Primary Care	Infrastructure, Leadership, Mentoring, Training	To present a position paper to guide development of a strategic planning process	IF academic leaders understand the research process and the types of infrastructure services and skills required to support a successful independent investigator, THEN organisations can identify experienced investigators willing to support each other and to mentor others
26. Nuyens (2007) [15]	International	Health and Health Services Research	Prioritisation	To look at major issues emerging from countries’ experiences in setting priorities during the past 15 years and at the challenges still to be addressed	IF national organisations institute a bottom-up approach to the generation of research priorities, THEN a context-sensitive and culturally sensitive process of priority-setting occurs
27. O’Byrne & Smith (2011) [58]	United Kingdom	Nursing	Facilitation, Infrastructure, Leadership, Mentoring, Networks, Prioritisation	To identify models used to provide local research opportunities and thereby develop research capacity and capability in clinical nurses in the United Kingdom	If organisations prioritise expansion of research initiatives for nurses and allocates resources for an accompanying infrastructure, THEN organisations achieve successful RCB
28. Pickstone et al. (2008) [59]	United Kingdom	Allied Health	Funding	To describe the nature of RCB in allied health professions and to explore the vision of RCB using the United Kingdom as an example	IF professionals receive sustained targeted funding to release them to undertake research, THEN professionals are able to resist workload pressures
29. Priest et al. (2007) [60]	United Kingdom	Nursing	Networks	To explore nursing lecturers’ RCD through their engagement as co-researchers in a larger case study project	IF organisations identify a specific person as a research contact, THEN staff interested in research involvement feel able to approach that person
30. Raghunath et al. (2004) [61]	United Kingdom	Primary care	Funding, Networks, Training	To explore the meaning, understanding, usefulness and reality of multidisciplinary research in primary care and provide examples	IF external assessment provides definable indicators of success, THEN organisations are able to demonstrate accountability and value for money
31. Ried et al. (2005, 2006, 2007) [62, 63, 64]	Australia	Primary care	Networks, Training	To understand the background and skills of the membership and to tailor South Australian Primary Health Care Research Network (SARNet) services to members’ needs	IF organisations utilise a whole system approach to RCB, THEN diverse individuals are encouraged to participate in research activities
32. Sarre & Cooke (2009) [65]	United Kingdom	Primary care	Infrastructure, Leadership, Training	To provide practical support to primary care organisations through the development of indicators against which to plan and measure progress of RCD at an organisational level	IF RCD occurs at different structural levels, including change and sustainable development in individuals, teams and organisations, THEN it can demonstrate clear links to the effectiveness and quality of healthcare organisations in improving health and well-being
33. Segrott et al. (2006) [66]	International	Nursing	Facilitation, Prioritisation, Training	To report a critical overview of nursing RCD in academic departments, major barriers to RCD, and capacity-building strategies from the literature, and to examine the wider context within which capacity-building takes place	IF departments have a flexible approach to research activities, THEN researchers are given the creative space to pursue their own research interests alongside core research priorities
34. Stephens et al. (2011) [67]	United States of America	Health and Health Services Research	Funding, Leadership	To synthesise and share what has been learned about RCB to help organisations and institutions develop and enhance their ability to plan and conduct health services research and obtain funding for their research	IF organisations secure departmental and institutional leadership support for capacity-building activities, THEN this facilitates future research activities IF research leaders demonstrate how a department/organisation’s existing experiences can be used to leverage and build an interdisciplinary team in health services research, THEN potential participants become less sceptical about the value of health services research
35. Van Weel & Rosser (2004) [68]	International	Primary Care	Networks, Training	To summarise World Organisation of Family Doctors (Wonca) conference discussions and present recommendations proposed by conference attendees from 34 countries	IF research teams display research achievements to policy-makers, health funders, and academic leaders, THEN policy-makers and others have a greater perception of the relevance of that research IF a tight link is created between clinical practice and a research environment, THEN clinicians and policy-makers will perceive the greater relevance of research to clinical practice
36. Whitworth et al. (2012) [3]	United Kingdom	Allied Health	Facilitation, Funding, Leadership, Mentoring, Networks, Training	To outline a comprehensive model developed and successfully implemented by speech and language therapists in North East England	IF organisations acknowledge the developmental stage at which the practitioner is positioned, THEN organisations can arrange suitable pathways into the research pathway

*LMIC*s low- and middle-income countries, *NIH* National Institutes for Health, *RCB* research capacity-building, *RCD* research capacity development, *RCS* research capacity strengthening

NB. For a more complete list of If-Thens see Additional file 1

^a^Citation pearls – key articles

Given that the aim of the project was interpretative and explanatory it was not considered necessary to comprehensively sample every possible RCD model, framework or theory. Instead, we were looking to construct theory and therefore employed a purposeful sampling approach described as ‘intensity sampling’. In the context of a research synthesis, intensity sampling involves selecting papers that are “*excellent or rich examples of the phenomenon of interest, but not highly unusual cases… cases that manifest sufficient intensity to illuminate the nature of success or failure, but not at the extreme*” [32]. This method of sampling appears particularly appropriate given that the intent of realist synthesis is to delve into inconsistencies of evidence in order to build programme theories to offer to policy-makers [29]. Sampling was operationalised through initial selection of the 10 citation pearls and, subsequently, through selection of articles that specifically sought to theorise or conceptualise RCD.

### Literature classification

The final list of included studies comprised 36 theoretical and conceptual papers (Table 2). The included literature reflected a wide range of environments and settings within which RCD might take place. The applied context for this synthesis meant we were particularly interested in United Kingdom-based studies, although Australia and the United States were particularly well represented. Primary care as a context was particularly prominent. Reasons for this may be temporal, with acute hospital infrastructures being at a later stage of development than primary care counterparts, related to saleability, with primary care organisations and networks being more able to facilitate actionable change, or may relate to external developments and priorities. We also identified papers that described North–South partnerships as exemplars of collaborations or networks.

### The focus of this paper

This paper focuses on 36 conceptual and theoretical papers (Table 2), including papers containing some form of framework or model of RCD. Models and frameworks were handled in the same way and were deconstructed into their original elements (in the form of If-Then statements). Thus, hypothesised relationships were not privileged within the original deconstruction but emerged naturally from the synthesised data.

A process of discussion and consensus led to identification of eight overarching programme theories, identifying mechanisms triggered across multiple (i.e. at least more than one) macro contexts (Table 3). Several programme theories recognise RCD as a collaborative effort facilitating academic engagement in health systems. Therefore, for example, the ‘more than sum of parts’ includes valuing and recognising as an asset, the contribution that each individual brings to the partnership, enabling discussion and joint thinking, access to different networks, and dividing workload across disciplines. This promotes respectful and meaningful discussion that can lead to trust and on-going dialogue and can be underlying mechanisms throughout diverse interventions, for example, in priority-setting, grant writing groups, communities of practice, knowledge transfer partnerships and doctoral training networks. Additionally, the resulting outcome provides a new context that can activate other mechanisms, for example, ‘coproduction of research’ and ‘learning by doing’. Thus, the outcome of one mechanism can produce the context to stimulate mechanisms in others. Reviewing the programme theories in Table 3 reveals that they form chains of ‘context (C) – mechanism (M) – outcome (O)’, where the outcome of one part of the chain shapes a subsequent context within which to stimulate the mechanism in others in the RCD programme. Similarly, ‘liberating talents’, ‘learning by doing’ and ‘releasing resources’ form chains, which may be usefully harnessed when developing fellowship and secondment interventions, for example.

**Table 3:** Overarching programme theories for Re:CAP (Expanded)

‘Label’	Elements of programme theory	Lines for further inquiry from mid-range theory	Example of source data
	*Research capacity will be effected if…*		*“This was what we achieved….” “This is what I think….”*
**By Activity**			
PT1. ‘Exceeding the sum of the parts’	Individuals/organisations/networks realise a contribution that they are unlikely or less likely to achieve in isolation	Social Network and Organisational Theory Community of Practice [69] Social Capital	“*Building research capacity is a complex challenge and needs to be thought of as a holistic process. Each constituent part is vital to the success of the whole, and is inextricably interlinked with all the others – a gestalt-like phenomenon in which the whole is greater than the sum of its parts*” [70]
PT2. ‘Learning by doing’	Individuals/organisations prototype or practise activities required for subsequent full engagement, and sequentially learn through cycles of reflection	Experiential Learning Model [55, 61] Learning Organisation	“*‘Learning by doing’ approaches, usually in the form of developmental or seed grants, hands-on training in ongoing research programmes or mentorship programmes, are effective approaches that complement academic degree offerings. They are also most appropriate for building capacity on the ‘demand’ side so that those who use research findings understand and appreciate their value in improving health outcomes*” [1]
		Social Capital Learning Organisation	“*Training should be and should remain one of the central foci when partnerships are awarded. Training young scientists in the context of such ongoing projects is irreplaceable (so-called ‘learning by doing’) and leads to a rapid acquisition and development of research skills and experience*” [57]
PT3.1 ‘Liberating the talents’	Individuals/organisations release the dormant potential of their skills and experience	Bourdieu’s Theory of Practice 1977	“*A more focused approach can accelerate progress in building capacity and allows researchers and teachers to develop their ‘natural talents’*” [70]
PT4. ‘Releasing resources’	Resources provided to overcome individual/organisational inhibition and act as a focus for activity, and information is freely shared about these opportunities	Lewin Model of Change (Unfreeze/Change/Freeze) Social Capital	“*Evidence of resource investment from the organisation to support pump-priming of research, e.g. research support sessions, pump-priming money for pre-protocol or pilot work*”
PT5. ‘Coproducing knowledge’	Individuals/organisations share ideas and knowledge development through networks and partnerships	Beresford [71] Coproduction [72, 73] Social Change	“*Engaging other stakeholders – such as service users, community members, health practitioners and policy-makers – is helpful for setting realistic goals, meeting local priorities and addressing resource issues. This requires extensive participation and hence more resources*” [74]
			“*A new kind of production of knowledge is emerging. This new model of knowledge production is called ‘Mode 2’ ...Mode 2 knowledge production is characterized as multi-professional driven, allowing ideas and knowledge to be generated and reflected upon within research groups which combine heterogeneous skills and experience. Within Mode 2, research groupings change from project to project and tend towards non-hierarchical, networking arrangements*” [52]
**Symbolic**			
PT6. ‘Feeling that you are making a difference’	Individuals/organisations perceive that research has an impact on health/wealth/knowledge creation/tackling inequalities	Social Change	“*Both nurses had never been involved in research before but were actively interested in taking part as they perceived that the research process would directly benefit patients in the short and long term, as well as giving them the opportunity to learn about research through a problem-based approach*” [61]
PT7. ‘Modelling positive behaviours’	Individuals observe the positive impact of involvement in research by others in the organisation		“*The best aspect of the workshop was having access to senior GP academics – as role models – and meeting early career researchers— as reassurance*” “*Role modelling from academics and research-minded registrars was influential. It was good to discover that academics were not scary!*” [75]
PT8. ‘Signalling importance and making research core business’	Individuals perceive that involvement in research is a valid activity in relation to competing priorities within the organisation	Social Norms	“*Some saw next stages as about consolidation, making sure that gains were hard-wired in and that senior managers themselves were proactive, for example in operationalizing research objectives through the appraisal system*” [76] “*The Office of Research staff …do not become involved in graduate student education or research presentation materials. The support services provide instrumental assistance and convey a clear message about the importance of preparing competitive grant applications*” [49]

A major finding from the qualitative synthesis and analysis was that many activities fulfil an emblematic (symbolic) role in signalling the importance of RCD within the organisations, networks or teams. Therefore, for example, the ‘protected time’ or ‘buy out from other responsibilities’ interventions initially seem to serve an instrumental role in freeing staff from their other duties in order to participate in research. On closer examination, however, it is clear that such activities are equally important in demonstrating to staff within an organisation that research is important and should be considered an organisational/system priority. Similarly, the development of small funding schemes in research networks or organisations, engagement in writing workshops and the promulgation of mentorship schemes assume significance beyond their monetary value in signalling that research is valued and hence an activity in which it is legitimate for staff to engage. A common trigger for all mechanisms is ‘making a difference’, which can stimulate motivation in stakeholders across the RCD programme, and can be seen as a community building exercise.

### Validation and refreshment of programme theories

Citation searches for the 10 citation pearls in December 2017 identified a further 10 systematic reviews published between 2014 and 2017 that met our original inclusion criteria (Table 4). Collectively, these covered all the RCD activities (Box 1) and reflected the diversity of contexts identified in the original dataset (e.g. high- (*n* = 5) and low- and middle-income countries (*n* = 5); allied health [33], nursing [34], public health [35], and health and health services research (*n* = 6), etc.). A new addition was in the emerging field of knowledge translation [36]. Again, the papers represented all the identified activities of RCD (Box 1), yet, since they were all reviews, they were more likely to describe multiple activities than the original dataset (Table 5).

**Table 4:** Reviews (2014–2017) used to validate programme theories

Author (Year) [Ref]	Context	Discipline	Aim of study	Indicative If-Thens
Borkowski et al. (2016) [33]	Systematic Review	Allied Health	To evaluate the evidence to increase understanding of factors that could influence AH research culture, in addition to identifying the enablers and barriers for AH professionals to conduct research	IF individuals are able to identify involvement in multiple research activities and identify research in their job descriptions, THEN they will have confidence in their research skills and abilities IF individuals identify a problem that needs changing, THEN they get involved in research activities
Dean et al. (2017) [77]	LMICs	Health and Health Services Research	To critically analyse collective health RCS effort regarding the level, type, cohesion and conceptual sophistication of the current evidence base	IF LMICs are to own research conducted in their own context, THEN reflexivity on the appropriateness of particular research for their country should be encouraged
Ekeroma et al. (2015) [78]	LMICs	Health and Health Services Research	To identify educational and other interventions that worked for clinicians, their characteristics and how they may have worked	IF novice researchers receive mentoring from experienced researchers, THEN novice researchers succeed in research activities IF individuals work together in designated research teams, THEN individuals are successful in research activities
Franzen et al. (2017) [48]	LMICs	Health and Health Services Research	To identify and critically examine the main approaches, strategies and trends in health RCD and consolidate key thinking in order to identify a more coherent approach	IF organisations develop and share a database of researchers, THEN researchers are able to network with each other IF organisations develop LMIC university research training capacity using ‘train the trainer’ programmes, THEN individuals feel able to engage with health research
Gagliardi et al. (2014) [36]	Systematic Review	Knowledge Translation	To review literature in management and social sciences and identify essential components of mentoring programmes that could be adapted for knowledge translation mentorship	IF a preliminary workshop is used to convey knowledge prior to mentoring, THEN mentoring can reinforce that prior knowledge
Huber et al. (2015) [79]	Systematic review	Health and Health Services Research	To review tools and instruments to aid health RCD initiatives in selecting appropriate tools and instruments for data collection within their respective context	IF national organisations pay attention to the sustainability of programmes and impact evaluation (e.g. parameters of patient care or societal aspects), THEN research meets the needs of the local populations
Kahwa et al. (2016) [80]	LMICs	Health and Health Services Research	To explore definitions, concepts, approaches and frameworks for RCB, to identify frameworks for evaluating RCB in healthcare, and to describe key challenges related to RCB in LMICs	IF leadership operates at an individual and a team level, THEN senior researchers support junior researchers and champion the development of institutional supports for research (including protected time for research) IF organisations offer ongoing training, mentoring and supervision, sharing of skills and expertise as well as providing opportunities to apply acquired skills, THEN individuals develop research skills, self-assuredness and a positive attitude towards doing and using research IF research questions are generated in consultation with users (practitioners and other service providers, and policy-makers), THEN researchers produce research that is relevant to prevailing health issues and concerns IF organisations support appropriate dissemination, THEN this enhances the social impact of research and ensures that it effectively influences practice IF organisations establish essential research structures and provide opportunities to apply and extend knowledge and skills gained into practice, THEN organisations can achieve continuity and sustainability IF organisations involve academic and management staff to supervise and manage projects, provide protected time for research, create research positions, and enhance knowledge about and access to research funding opportunities, THEN organisations support participation in research/related capacity-building initiatives IF researchers develop partnerships and linkages and thus expand intellectual and social capital, THEN this facilitates the exchange of research skills, knowledge and expertise
Lode et al. (2015) [34]	Systematic Review	Nursing	To identify and evaluate evidence of clinical nurses’ RCB in practice	IF organisations strengthen nurses’ belief in the value of research and of research teams, THEN nurses will participate in research activities
Mugabo et al. (2015) [81]	LMICs (Africa)	Health and Health Services Research	To contribute to RCD efforts by providing insights from different approaches that could be applied to other locations and to encourage more complete reporting of such initiatives	IF organisations develop a strong institutional infrastructure, THEN organisations succeed in securing research funding IF experienced researchers mentor novice researchers on publications, THEN novice researchers are able to publish their research in high profile international journals
Norton et al. (2016) [35]	Systematic Review	Public Health	To identify evaluated strategies used by organisations and programme developers to build the programme evaluation capacity of their workforce, and to describe success factors and lessons learned	IF organisations demonstrate that evaluation is an organisational focus or priority, THEN individuals will consider involvement in evaluation to be important IF organisations embed evaluation into work processes through policy and procedures that uphold evaluation expectations, THEN organisations demonstrate strong evaluation leadership

*AH* Allied Health, *LMICs* low- and middle-income countries, *RCB* research capacity building, *RCD* research capacity development, *RCS* research capacity strengthening

**Table 5:** Activities identified in reviews (2014–2017)

Author (Year) [Ref]	Research capacity development activities included
Borkowski et al. (2016) [33]	Infrastructure, Leadership, Mentorship, Training
Dean et al. (2017) [77]	Infrastructure, Networks, Training
Ekeroma et al. (2015) [78]	Mentoring, Networks, Training
Franzen et al. (2017) [48]	Leadership, Mentoring, Networks, Training
Gagliardi et al. (2014) [36]	Mentoring
Huber et al. (2015) [79]	Infrastructure
Kahwa et al. (2016) [80]	Funding, Infrastructure, Leadership, Mentoring, Networks, Training
Lode et al. (2015) [34]	Funding, Leadership, Networks, Training
Mugabo et al. (2015) [81]	Infrastructure, Mentoring, Training
Norton et al. (2016) [35]	Infrastructure, Leadership, Training

The identified reviews confirmed the original programme theory, adding a nuanced understanding of many RCD activities. However, the new dataset did not identify any new strands of programme theory, possibly indicating that theoretical saturation had been reached. Evaluation, debatably an RCD activity in its own right, emerged as increasingly prominent in the recent literature. However, evaluation was considered to be a subtext to all the other activities and was not included as an additional activity. Future conceptual models should ensure that they feature evaluation, albeit to be handled differently from other activities.

## Discussion

Many governments and global partnerships invest considerable funds to support RCD in healthcare, and it is a moral and ethical imperative to develop, shape and evaluate such activity [37] in order to plan and attain the desired effect, and to justify continued funding [16]. This unique realist synthesis of the conceptual literature on RCD has uncovered mechanisms that operate beneath such activities which, we suggest, can function across and within different structural levels with an emphasis of meaningful societal impact. We suggest that the programme theories developed here might help to plan and demonstrate cohesion and alignment across structural levels. Several authors recognise that RCD activities need to take place concurrently at a number of different levels [1, 8, 13], with many calling for a ‘whole systems’ approach to RCD [3, 19]. We propose that the programme theories developed here could act as a guide for application across the diverse individual, organisational and network levels in order to promote synergy and ensure RCD activities ‘pull’ in the same direction.

Social change is the ultimate outcome for RCD [8]. The programme theories presented here, particularly those described as ‘symbolic’, can provide visible mechanisms of how RCD might influence culture, leadership and motivation. Many of these mechanisms have foundations in theories of social change and social capital, and provide an explanation of how interventions that adopt such an approach can engender a research culture within organisations and networks, and reciprocity and leadership in individuals.

Our programme theories resonate with others that explain how research activity can promote impact in healthcare organisations and communities [38, 39, 40]. Whilst training in research traditionally includes research methods, data collection and analysis skills, the co-production programme theory suggests diverse skills of cross boundary working, negotiation and creative practices in knowledge production [39]. Researchers using a co-productive approach are more likely to align research with stakeholder and organisational objectives [1] and form dynamic partnerships using assets from different organisations and networks [38] to make a difference.

Under what circumstances are RCD interventions most likely to achieve their intended effect? Our analysis has identified several principal components from a theoretical perspective:

1. RCD interventions may act as a catalyst for releasing potential research energies from within individuals and organisations. This is most clearly seen in the programme theories that relate to ‘Exceeding the sum of the parts (PT1)’, ‘Liberating the talents (PT3)’ and ‘Releasing resources (PT4)’. The implication is that, without such triggers, the organisation and individuals remain essentially inert or slow moving with regard to their engagement in research activities.
2. RCDs must meet criteria for observability, meaning that current and potential participants must be able to perceive potential and actual benefits from their involvement. This is most clearly seen in the collective programme theories labelled as symbolic (or emblematic), i.e. ‘Feeling that you are making a difference (PT6)’, ‘Modelling positive behaviours (PT7)’ and ‘Signalling importance (PT8)’. However, it is additionally present within the ‘Learning by doing (PT2)’ programme theory, where trainers and trainees receive almost synchronous confirmation of personal growth and skills acquisition.
3. RCDs must secure the engagement and commitment of their stakeholders and beneficiaries. Such commitment may be overtly signalled through explicit strategies or statements on research, through the celebration of achievements and through the provision of protected time from the demands of competing activities (‘Signalling importance PT8). It can also be secured by co-creation opportunities through ‘Releasing resources (PT4)’ and ‘Coproducing knowledge (PT5)’ with added opportunities for ‘Feeling that you are making a difference (PT5)’.
4. Possible linkages and C-M-O chains are emerging, where the outcome of one mechanism stimulates another within the programme architecture, and can act as leverage within it. For example, the mechanisms that are symbolic (PT6–8) may nurture a research culture that acts as a backdrop to other activities (P1–7). Variation in this cultural backdrop can be conceived as the effect of a dimmer switch [41] by which the range of outcomes of the symbolic mechanisms have correspondingly greater or lesser influence on associated RCD activities. The more evident a research culture, the more assets/resources that culture is able to bring to the RCD architecture and, correspondingly, the more power released within the dimmer switch to stimulate a range of mechanisms across the programme.

Thus, the findings from this realist synthesis of the conceptual RCD literature offer a starting point for constructing a RCD framework shaped by these programme theories. Future work should include exploration, elaboration and iterative refinement of these programme theories through exploration with other theory (some suggestions are included in Table 3), and testing against empirical data from intervention studies [42, 43].

### Strengths

We have taken a first step in developing components of an overarching theory to determine what works for whom to accomplish ‘more research done well’. Searches were conducted across a wide range of databases and were supplemented by exhaustive reference checking and citation tracking. The 36 conceptual and theoretical papers we identified are derived from diverse settings and describe RCD activities at multiple levels, strengthening confidence in the identification of candidate mechanisms. All major RCD activities are identifiable in both the included set of papers and in the validation set of recent reviews with which we tested our initial findings. Our realist-based approach offers an opportunity for a more nuanced understanding of how interventions might work and, indeed, in understanding circumstances in which they may not, which goes beyond the mere presence or absence of a specific intervention, a serendipitous bundle of collective interventions or a tailored package of synergistic initiatives.

### Limitations

The systematic mapping process was subject to time and resource constraints and was primarily conducted by one investigator. Validation of a 20% sample was performed by the other two investigators to sensitise team members to the characteristics of the evidence base. The team reached a consensus on what should be recognised as constituting a model, theory or framework but did not distinguish between these three contested terms. The articles studied for the presence of programme theory were purposively sampled from a wide range of candidate studies and were selected to represent diverse settings and contexts and because of the perceived richness of their data.

Our preliminary findings have already been shared with a group of nine National Health Service organisations who meet to promote organisational development in RCD, called ACORN (Addressing Capacity in Organisations to do Research Network) [44]. Individuals were able to comment on the extent to which these programme theories fit with their practical experience of RCD.

Potential implications, based upon our theoretical frameworks, for those planning RCD at different contextual levels are given in Box 2.

## Conclusions

Realist evaluation approaches are increasingly common when evaluating specific interventions in RCD [45, 46, 47]. Other authors are further using innovative literature review methods in order to explore development strategies for RCD [48]. We believe that this is the first time that the innovative approach of realist synthesis has been applied to conceptual papers on RCD in order to isolate the underpinning RCD programme theories. The value of this approach is in drilling down beneath the activities of a programme to identify the mechanisms that are deployed therein.

This review found that, collectively and individually, RCDs engage with multiple defined programme theories to achieve their potential impact. Such programme theories have a role in developing new RCD interventions, in modifying existing initiatives, and in creating a comprehensive evaluation framework against which to measure achievements. We have been able to tentatively explore how C-M-O might link to develop ‘trigger’ chains and speculate how symbolic mechanisms may link to interventional ones. This needs to be explored further within intervention studies. Our initial work requires further development to extend the analysis and thus cover a full range of RCD interventions, mapping both activities and evaluation measures by intervention, stage in the development lifecycle, and by programme theory.

Our investigation represents an overt attempt to capitalise on the utility of realist synthesis for the specific tasks of theory generation and subsequent exploration of potential mechanisms. Gough et al. [28] characterise the potential contributions of synthesis in general in terms of generating, exploring and testing (G-E-T) of theory. A subsequent stage of this project is therefore to test these emergent concepts with reference to empirical studies, either relating to research capacity as a composite activity or to the individual interventions by which we characterise research capacity activities. Planned outcomes from the subsequent stages of this project include identifying which mechanisms are associated with specific types of intervention and the development of an evaluation framework with which to assess the achievements of general RCD programmes and their constituent interventions.

Our novel evidence-based model identified 36 conceptually rich papers relevant to RCD. Although we acknowledge that other papers hold the potential to inform our theory development, particularly in relation to the characteristics of individual interventions, we believe that we have identified the more common and significant programme theories that relate to RCD. We anticipate that further exploration will reveal a point of theoretical saturation and may help in identifying some of the nuances or, indeed, disconfirming cases, associated with specific RCD initiatives. Ultimately, we hope that the conceptual framework presented in this paper will contribute to the demonstration of long-term outcomes in health, wealth and knowledge as commissioners and service providers work together to increase RCD.

## Box 1 Activities undertaken in research capacity development (RCD) (from Re:CAP [10])

1. Prioritisation: Developing research priorities from consensus views of informed participants.
2. Mentoring: where an experienced, highly regarded person (the mentor) guides another individual (the mentee) in the development and examination of their own ideas, learning, and personal and professional development.
3. Leadership: the process of influencing group activities towards the achievement of RCD goals.
4. Research facilitators: individuals whose role is explicitly to promote and enable the conduct of a research by those with limited research experience.
5. Training: interventions that aim to increase skills and knowledge.
6. Funding to develop RCD including bursaries and fellowships.
7. Networks and collaborations: structures and functions that support people to work together to improve knowledge transfer, innovation, a research process or an output.
8. Infrastructure: a range of activities used to enhance support of RCD.

## Box 2 Potential implications when planning RCD

**Funding bodies**

- Develop research priority-setting mechanisms to release resources to fund research that can ‘make a difference’. Priorities should be agreed between stakeholders to co-produce knowledge that will have an impact on health and wealth.
- Develop funding opportunities to support ‘learning by doing’ opportunities for individuals, to compliment more formal research training.
- Fund career pathways and liberate talents through actively seeking individuals with potential, and fund coaching and mentorship to maximise this.
- Fund appointments between healthcare and academic organisations to support partnerships that exceeds sum of parts and co-production of research, and provide positive role models.
- Develop funding calls to a release resource and signal importance of research activity within healthcare organisations. For example, through a matched funding model in large research programmes, and protected time agreed with managers in ‘learning by doing’ opportunities.
- Fund novel methods of research dissemination that promote action in clinical and healthcare practice so that the research findings can make a difference, and signal importance of implementing research knowledge into practice.

**Healthcare organisations**

- Signal the importance of research activity within the organisation through job descriptions, mission statements, training and R&D strategies. Support business plans in order to release resources that will include protected research time and ring-fenced research resources.
- Recognise and celebrate positive research behaviours in clinical academic staff, managers and services through award schemes and communication channels.
- Enable mentoring and coaching schemes to be undertaken in their organisation in order to release potential talent and support learning by doing activities.
- Seek and support ‘learning by doing’ programmes as well as more traditional research training opportunities.
- Develop a needs and assets register to recognise and liberate talent.
- Develop a sense of ownership and commitment to research activity, through co-creation of research ideas, observable instances of quick wins and impact success stories, to demonstrate research that makes a difference.
- Work proactively in partnership with other organisations, networks and academic institutions to maximise synergies, coproduction, ‘learning by doing’ opportunities.

**Individuals:**

- Recognise the personal, organisational and long-term benefits from their own involvement in research to make a difference and to demonstrate positive research behaviours.
- Seek and use leaning by doing opportunities.
- Support release of their own talents, and that of others around them. Be both a mentor and a mentee. Use coaching opportunities to release their own potential.
- Work with managers to negotiate protected time to signal importance of research alongside practice.
- Develop skills to support coproduction of research, and plan research activity that has an impact on practice to make a difference.
- Are able to develop skills and knowledge through practical involvement in research activities, including alignment to organisational objectives.

## Additional file

Additional file 1: The 36 studies describing conceptual models, frameworks or theory for research capacity development. (DOCX 35 kb)

## Abbreviations

KM: knowledge mobilisation
RCD: research capacity development
Re:CAP: Research Capacity – A scoping review to identify the evidence-base for Research Capacity development in health and social care
