# Supplementary material for: Uncovering the mechanisms of research capacity development in health and social care: a realist synthesis
Source: Health Res Policy Syst. 2018 Sep 21;16:93. doi: 10.1186/s12961-018-0363-4 (PMC6150992; doi:10.1186/s12961-018-0363-4)
Supplement: Supplementary file 1 — The 36 studies describing conceptual models, frameworks or theory for research capacity development. (DOCX 35 kb) [file 12961_2018_363_MOESM1_ESM.docx]

**Additional file 1 - 36 Studies describing Conceptual Models, Frameworks or Theory for Research Capacity Development**

| **Author (Year) [Ref Id]** | **Context** | **Discipline** | **Included Research Activities** | **Indicative IF-THENs** |
| --- | --- | --- | --- | --- |
| 1. Albert & Mickan (2003)[4] | Australia | Primary Care | Training | IF research ideas and implementation strategies are discussed and translated across several organisational contexts and cultures THEN research influences practice |
| 1. Breen et al (2005)[21] **[Pearl]** | South Africa | Public Health | Leadership; Networks; Resources; Training | IF intended adopters share an understanding of the need for and purpose of the research THEN they are better prepared for the outcomes, and diffusion and adoption is enhanced.  IF investment is inadequate and incentives inappropriate THEN organisations are unable to sustain RCD beyond the lifespan of a specific project. |
| 1. Coen et al (2010)[31] | Canada | Health and Health Services Research | Infrastructure, Leadership, Networks, | IF organisational leaders develop an appropriate organisational research culture THEN members collaborate on research.  IF organisational leaders develop an appropriate organisational research culture THEN members are stimulated to be creative and innovative.  If researchers share a particular research identity THEN researchers acquire a sense of belonging or a ‘ready-made affinity group’.  IF researchers share a sense of belonging THEN researchers collaborate on research. |
| 1. Condell & Begley (2007)[8] | International | Nursing | Funding, Leadership, Training | IF organisations engage in dynamic RCD activities THEN organisations can achieve sustainability and ultimately effect social change. |
| 1. Conn et al (2005)[49] | USA | Nursing | Funding, Prioritisation | IF faculty determine (and own) the research interest groups and priorities THEN they are motivated to engage in the research process.  IF a grant specialist focuses on funding processes and procedures THEN the faculty can focus on the research plans.  IF staff in grant support services are not involved in graduate student education or research presentation materials THEN this conveys a clear message about the importance of preparing competitive grant applications.  IF an organisation frequently communicates about grant activity THEN the organisation cultivates an environment that is conducive to high research productivity |
| 1. Cooke & Green (2000)[50] | International | Nursing Research | Prioritisation, Training | IF nurse educators are encouraged to pursue further qualifications, particularly higher degrees, THEN teaching staff feel able to engage in research activity |
| 1. Cooke et al (2005)[9] **[Pearl]** | UK | Primary Care | Networks, Training | IF organisations develop appropriate skills, and confidence in their staff, through training and creating opportunities to apply skills THEN they will feel able to participate in research activities.  IF organisations support research 'close to practice' THEN stakeholders perceive that research is useful  IF organisations develop linkages, partnerships and collaborations THEN stakeholders exchange, develop and enhance research skills and practice knowledge.  IF organisations develop linkages, partnerships and collaborations THEN organisations can build up intellectual capital (knowledge) and social capital (relationships)  IF organisations build up intellectual capital (knowledge) and social capital (relationships) THEN their ability to do research is enhanced.  IF different groups and individual researchers build up trust among themselves THEN groups and individuals can enhance information and knowledge exchange.  IF organisations ensure appropriate dissemination of research outputs THEN research will achieve maximal impact.  IF research funders include continuity and sustainability in funding provision THEN organisations can maintain and continue newly acquired skills and structures to undertake research.  IF organisations set up structures and processes to enable smooth and effective running of research projects THEN this reduces barriers to participation and enable skills and enthusiasm to be developed. |
| 1. Cooke et al (2015)[12] | UK | Health and Health Services Research | Funding, Leadership, Networks, Prioritisation, Training | IF research networks engage in ongoing dialogue with relevant stakeholders using appropriate methods and negotiation THEN research is aligned with population priorities  IF research networks identify 'Needs-led meaningful' research projects THEN research is considered timely  IF research networks harness flexible resources (people, funds, skills) THEN research can be responsive  IF research leadership is responsive and transformative THEN research can be co-produced. |
| 1. Del Mar & Askew (2004)[18] **[Pearl]** | Australia | Primary Care | Funding, Networks, Training | IF governments have family medicine research on their agendas (as shown by funding for RCB and for research activity itself) THEN governments send a clear message to clinical and academic communities that family medicine research is important and worthy of support. |
| 1. Edwards et al (2009)[51] | LMICs | Nursing Research | Funding, Mentoring, Training | IF researchers engage with post-graduate training including post-masters and post-doctoral fellowships THEN researchers can successfully apply newly acquired research skills  IF researchers are given opportunities to work alongside senior researchers, both on-site and by distance THEN they can discuss ways to balance research with teaching, clinical and administrative demands. |
| 1. Farmer & Weston (2002)[19] **[Pearl]** | Australia | Primary Care | Funding, Mentoring, Networks | IF research funders employ a whole system approach providing funding and resources at multiple levels THEN practitioners can enter the system at an appropriate level, and then progress to a higher level of research capacity  IF research funders accommodate diversity THEN practitioners develop research interests in topics of ongoing personal interest.  IF research funders provide protected time for research THEN individuals participate in research.  IF university departments and practices jointly fund a research post THEN collaboration takes place across organisations  IF organisations make provision for mentoring THEN participants benefit from personal attention, guidance, motivation and feedback from mentors  IF research funders establish networks THEN participants have opportunities for research collaborations |
| 1. Fenton et al (2001, 2007) [52][53] | UK | Primary Care | Networks | IF researchers demonstrate socialization, teamwork and openness THEN researchers resist the tendency towards groupthink and open up opportunities for the exchange of ideas and knowledge  IF researchers identify with a network THEN researchers are likely to recognise opportunities offered by participation |
| 1. Fitzgerald et al (2003)[54] | USA | Nursing | Mentoring, Networks | IF healthcare professionals are included as an integral part of the research team THEN healthcare professionals receive mentoring in many aspects of the research process  IF healthcare professionals are pulled away from their clinical unit to engage in research responsibilities THEN other staff may resent the intrusion or see involvement in the project as frivolous when they are left with more work or without ready access to consultation. |
| 1. Gadsby (2011)[16] | LMICs | Health and Health Services Research | Funding, Networks, Training | IF donors support individual capacity development at the expense of system capacity development THEN individuals from LMICs leave for better jobs elsewhere |
| 1. Golenko et al, (2012)[55] | Australia | Allied Health | Infrastructure, Leadership | IF line managers don’t support RCD THEN, in spite of organisational policies and procedures, RCD fails  IF RCB is built into people’s jobs THEN RCD is successful  IF RCB commands good leadership support THEN RCD is successful  *IF governance structures are in place with coordinated support to make sure it happens appropriately THEN staff are motivated to participate in research.*  *IF incentives are provided for staff to participate in the research, and thus benefit their career path, THEN staff are motivated to participate in research.*  *IF staff are supported from a staff time perspective to do research THEN staff are motivated to participate in research*  *IF staff receive recognition for research participation THEN staff are motivated to participate in research.*  *IF organisations run evidence-based journal clubs or groups within your organisation, THEN people with interest, but not experience, can spend time with people who know how to do it and be encouraged* |
| 1. Green et al (2007)[56] | UK | Nursing Research | Leadership, Networks, Training | IF researchers form alliances between novice and experienced researchers THEN organisations achieve a balance between capacity development and leading edge development  IF researchers engage widely with in-house postgraduate courses THEN researchers improve their academic profile of researchers is increased  IF researchers engage widely with in-house postgraduate courses THEN this has a positive impact on the department’s research culture. |
| 1. Jenerette et al (2008)[6] **[Pearl]** | U.S. | Nursing Research | Networks | IF partners have respect for each other’s strengths THEN collaborations are more successful  IF partners demonstrate effective communication and are sensitive to the history and unique characteristics of the partnering institution as well as its population THEN investigators successfully complete projects on time and deliver subsequent presentations and publications |
| 1. Johnson et al (2005)[20] **[Pearl]** | Liberia | Health and Health Services Research | Infrastructure, Training | IF participant accepts the North South partnership THEN participant is ready to participate in research  IF participant perceives the salience of the North-South partnership THEN the participant is ready to participate in research  IF participant is receptive to communication mechanisms THEN participant is ready to participate in research  IF participant is ready to participate in research THEN they continue their ongoing participation in research  IF participants are ready to participate in research THEN their organisation sustains its research activities |
| 1. Jones et al (2003)[26] **[Pearl]** | Australia | Primary Care | Training | IF GPs perceive that they do not possess the necessary research skills THEN GPs are reluctant to engage in research |
| 1. Lansang & Dennis (2004)[1] **[Pearl]** | LMICs | Health and Health Services Research | Funding, Infrastructure, Mentoring, Networks, Training | IF the countries of the North offer masters', doctoral and postdoctoral training programmes to scientists from low-income and middle-income countries then these scientists can expand their competencies.  IF research funders promote "Learning by doing" approaches, such as developmental or seed grants, hands-on training in ongoing research programmes or mentorship programmes THEN practitioners are encouraged to participate in research  IF developing countries develop partnerships and networks with developed countries or other developing countries THEN their collective outputs are greater than the sum of their isolated efforts.  IF funders set up international centres of research excellence THEN these contribute sustainability and consistent quality to capacity building |
| 1. Levine et al (2013)[13] | USA | Health and Health Services Research | Funding, Infrastructure, Mentoring, Networks, Training | IF organisations develop good external and internal health services research partners THEN they can build research capacity  IF researchers have supportive senior administrators within their organization THEN they achieve grant success.  IF organisations receive external funds to build institutional capacity and conduct health services research THEN they can achieve successful RCB  IF organisations are able to build on or leverage larger organizational changes THEN they can achieve successful RCB |
| 1. Macfarlane et al (2005)[14] **[Pearl]** | UK | Primary Care | Funding, Infrastructure, Leadership, Networks, Training | IF networks provide small amounts of funding to GPs THEN GPs have an opportunity to undertake research activity  IF organizations produce a mission statement that acknowledges the value of research THEN GPs develop a research practice |
| 1. Mahmood et al (2011)[5] | Bangladesh | Health and Health Services Research | Funding, Prioritisation | IF organisations develop a monitoring and evaluation framework THEN donors do not exert an influence over organisational research priorities. |
| 1. Nchinda (2002)[57] | LMICs | Health and Health Services Research | Training | If young graduates are working or have worked as research assistants in ongoing research projects in their institutions THEN organisations can determine their suitability for doctoral level studies.  IF returning researchers learn new skills and techniques when training overseas THEN these researchers require access to appropriate equipment and resources when returning to their own institutions. |
| 1. North American Primary Care Research Group (2002)[27] **[Pearl]** | North America | Primary Care | Infrastructure, Leadership, Mentoring, Training | IF academic leaders understand the research process and the types of infrastructure services and skills required to support a successful independent investigator THEN organisations can identify experienced investigators willing to support each other and to mentor others |
| 1. Nuyens (2007)[15] | International | Health and Health Services Research | Prioritisation | IF national organisations institute a bottom-up approach to generation of research priorities THEN a context-sensitive and culturally sensitive process of priority setting occurs with resulting priorities being implemented at subnational levels. |
| 1. O’Byrne & Smith (2011)[58] | UK | Nursing | Facilitation, Infrastructure, Leadership, Mentoring, Networks. Prioritisation | IF senior nurses lead RCB initiatives THEN organisations achieve successful RCB  If organisations prioritise expansion of research initiatives for nurses and allocates resources for an accompanying infrastructure THEN organisations achieve successful RCB |
| 1. Pickstone et al (2008)[59] | UK | Allied Health | Funding | IF organisations manage the balance between profession-specific research and professional contribution to organisational research THEN this reconciles tensions between science push and service pull.  IF professionals receive sustained targeted funding to release them to undertake research THEN professionals are able to resist workload pressures |
| 1. Priest et al (2007)[60] | UK | Nursing | Networks | IF organisations identify a specific person as a research contact THEN staff interested in research involvement feel able to approach that person |
| 1. Raghunath et al (2004)[61] | UK | Primary care | Funding, Networks, Training | IF external assessment provides definable indicators of success THEN organisations are able to demonstrate accountability and value for money |
| 1. Ried et al (2005, 2006, 2007)[62,63,64] | Australia | Primary care | Networks, Training | IF networks offer opportunities for training and promote the use of research THEN the research culture of primary care organisations changes  IF organisations utilise a whole system approach to RCB THEN diverse individuals are encouraged to participate in research activities |
| 1. Sarre & Cooke (2009)[65] | UK | Primary care | Infrastructure, Leadership, Training | IF RCD occurs at different structural levels, including change and sustainable development in individuals, teams and organizations THEN RCD can demonstrate clear links to the effectiveness and quality of healthcare organizations in improving health and well‐being. |
| 1. Segrott et al (2006)[66] | International | Nursing | Facilitation, Prioritisation, Training | IF departments encourage proactive support for RCD THEN individuals engage in research development activities  IF organisations create an inclusive research environment THEN specific RCB interventions succeed  IF departments have a flexible approach to research activities THEN researchers are given the creative space to pursue their own research interests alongside core research priorities |
| 1. Stephens et al (2011)[67] | US | Health and Health Services Research | Funding, Leadership | IF organisations secure departmental and institutional leadership support for capacity building activities THEN this facilitates future research activities  IF research leaders demonstrate how department/ organization’s existing experiences can be used to leverage and build an interdisciplinary team in health services research THEN potential participants become less sceptical about the value of health services research |
| 1. Van Weel & Rosser (2004)[68] | International | Primary Care | Networks, Training | IF research teams display research achievements to policy makers, health funders, and academic leaders THEN policy makers and others have a greater perception of the relevance of that research.  IF a tight link is created between clinical practice and a research environment THEN clinicians and policy makers will perceive the greater relevance of research to clinical practice.  IF researchers establish an improved working relationship with the wider scientific community THEN researchers are able to demonstrate the higher scientific quality of their research.  IF research training and a career path are provided for clinical researchers THEN clinical researchers are able to see the value of a research career. |
| 1. Whitworth et al (2012)[3] | UK | Allied Health | Facilitation, Funding, Leadership, Mentoring, Networks, Training | IF Academics and Practice build up reciprocal Trust THEN this promotes co-operation and participation in joint research work  IF organisations acknowledge the developmental stage at which the practitioner is positioned THEN organisations can arrange suitable pathways into the research pathway. |

Key - RCB – Research Capacity Building; RCD – Research Capacity Development; RCS – Research Capacity Strengthening
